# Development of Useful Recombinant Promoter and Its Expression Analysis in Different Plant Cells Using Confocal Laser Scanning Microscopy

**DOI:** 10.1371/journal.pone.0024627

**Published:** 2011-09-09

**Authors:** Deepak Kumar, Sunita Patro, Rajiv Ranjan, Dipak K. Sahoo, Indu B. Maiti, Nrisingha Dey

**Affiliations:** 1 Department of Gene Function and Regulation, Institute of Life Sciences, Department of Biotechnology, Government of India, Nalco Square, Chandrasekherpur, Bhubaneswar, Orissa, India; 2 Kentucky Tobacco Research and Development Center (KTRDC), College of Agriculture, University of Kentucky, Lexington, Kentucky, United States of America; Purdue University, United States of America

## Abstract

**Background:**

Designing functionally efficient recombinant promoters having reduced sequence homology and enhanced promoter activity will be an important step toward successful stacking or pyramiding of genes in a plant cell for developing transgenic plants expressing desired traits(s). Also basic knowledge regarding plant cell specific expression of a transgene under control of a promoter is crucial to assess the promoter's efficacy.

**Methodology/Principal Findings:**

We have constructed a set of 10 recombinant promoters incorporating different up-stream activation sequences (UAS) of *Mirabilis mosaic virus* sub-genomic transcript (MS8, -306 to +27) and TATA containing core domains of *Figwort mosaic virus* sub-genomic transcript promoter (FS3, −271 to +31). Efficacies of recombinant promoters coupled to GUS and GFP reporter genes were tested in tobacco protoplasts. Among these, a 369-bp long hybrid sub-genomic transcript promoter (MSgt-FSgt) showed the highest activity in both transient and transgenic systems. In a transient system, MSgt-FSgt was 10.31, 2.86 and 2.18 times more active compared to the CaMV35S, MS8 and FS3 promoters, respectively. In transgenic tobacco (*Nicotiana tabaccum*, var. Samsun NN) and *Arabidopsis* plants, the MSgt-FSgt hybrid promoter showed 14.22 and 7.16 times stronger activity compared to CaMV35S promoter respectively. The correlation between GUS activity and *uidA*-mRNA levels in transgenic tobacco plants were identified by qRT-PCR. Both CaMV35S and MSgt-FSgt promoters caused gene silencing but the degree of silencing are less in the case of the MSgt-FSgt promoter compared to CaMV35S. Quantification of GUS activity in individual plant cells driven by the MSgt-FSgt and the CaMV35S promoter were estimated using confocal laser scanning microscopy and compared.

**Conclusion and Significance:**

We propose strong recombinant promoter MSgt-FSgt, developed in this study, could be very useful for high-level constitutive expression of transgenes in a wide variety of plant cells.

## Introduction

The overall strength of a promoter as well as its tissue-specific expression pattern depends on the combination of the spatial orientation of cis-elements and interaction with nuclear protein factors [1], [2]. Such interactions suggest that the cis-element-containing upstream activation sequence (UAS) plays an important role in determining promoter function [3]. Moving the DNA element (cis-factor) that binds a specific trans-factor from one promoter into a different promoter can result in a novel transcription model [4], [5], that can modulate the transcriptional activity and cell specific expression pattern of the promoter [6]. Several recombinant/synthetic promoters have been created earlier by (a) ligating the upstream activation sequence (UAS) of one promoter with the TATA box containing domain of another promoter; like tacI/tacII hybrid promoters [7], E4/E8 hybrid promoters [8] and chimeric plant promoter (Mac promoter) [9]; (b) placing desired cis-elements in conjunction with heterologous promoters [10]; (c) bringing together cis-elements from different promoters [11]–[13]. The strength and tissue specificity of chimeric promoters derived by incorporating sub-domains of the mannopine synthase (*mas2*) and octopine synthase (ocs) promoters were investigated earlier [14]. The Mac promoter, incorporating the *mas* region from +65 to −301 and the CaMV35S enhancer region from −90 to −941, has been reported to enhance the level of GUS expression by several folds as compared to the CaMV35S promoter [9]. Similarly, the strength and tissue specificity of Mac- and super-promoters were analyzed [14]–[16]. Novel plant transformation vectors incorporating the super-promoter were tested in transgenic tobacco and maize plants, and also transiently in maize protoplasts [16].

Risk of successive transformation by single promoter is that it might lead to silencing of transgenes in successive generations by homologous recombination [13], [17]–[20]. Use of different heterologous promoters is necessary during pyramiding (stacking) of genes in a single plant cell for developing a particular trait in plants. As in the case of developing β carotene (Provitamin A) enriched rice multiple genes coupled to different promoters were inserted in rice endosperm for engineering the concerned metabolic pathway/s [21].

Unique clustering of different plant-specific elements like ASF-1 (TGACG), ARR1AT (NGATT), Dof-1 (AAAG), WRKY (TGAC) AINTEGUMENTA (ANT), ATHB-9 and the GATA Box (GATA) among the *Mirabilis mosaic virus* sub-genomic transcript promoter (−356 to −125 of MS8) [22] and the TATA containing sub-domain of the *Figwort mosaic virus* sub-genomic transcript promoter (−151 to +31 of FS3) [23] sequences were identified using PLACE and PlantPAN databases [24], [25]. This finding prompted us to develop useful recombinant promoters by fusing different up-stream sub-domains of the MS8 promoter with different core promoter elements of the FS3 promoter.

In the present study, we have characterized a set of 10 unique pararetrovirus-based recombinant sub-genomic transcript promoters, and their expression activities were compared to that of the CaMV35S, MS8 and FS3 promoters in transient expression systems. An in-depth expression analysis of one of these hybrid promoters (MSgt-FSgt), which conferred the highest level of activity, was further studied in transgenic tobacco and Arabidopsis plants. Correlation between the MSgt-FSgt promoter-driven GUS activity and the uidA-mRNA level in transgenic plants was assayed. The interaction between the MSgt-FSgt promoter sequence and tobacco nuclear proteins was also analyzed by Electrophoretic Mobility Shift Assay (EMSA) and DNA Foot-printing. The cell specificity of MSgt-FSgt was compared to that of the CaMV35S promoter in different plant cells of the stem, leaf, and root using Confocal Laser Scanning Microscopy (CLSM). The recombinant MSgt-FSgt promoter reported here will be a valuable addition to the tools available for plant gene expression studies.

## Materials and Methods

### Materials

Restriction and modifying enzymes were purchased from Promega (Madison, WI USA), and were used according to the manufacturer's instructions. The Nytran membrane was obtained from Schleicher & Schuell (Keene, NH, USA). All fine chemicals, including MUG, X-gal, X-gluc, DEPC were purchased from Sigma-Aldrich (St. Louis, USA). Platinum high fidelity *Taq* DNA polymerase was obtained from Gibco-BRL (Maryland, USA). DNA methyltransferase *M.SssI* and *E.coli* K12 ER2925 were purchased from New England Biolabs, Beverly, MA, USA.

### Construction of expression vectors containing recombinant promoters for transient expression assay

**Figure 1:**
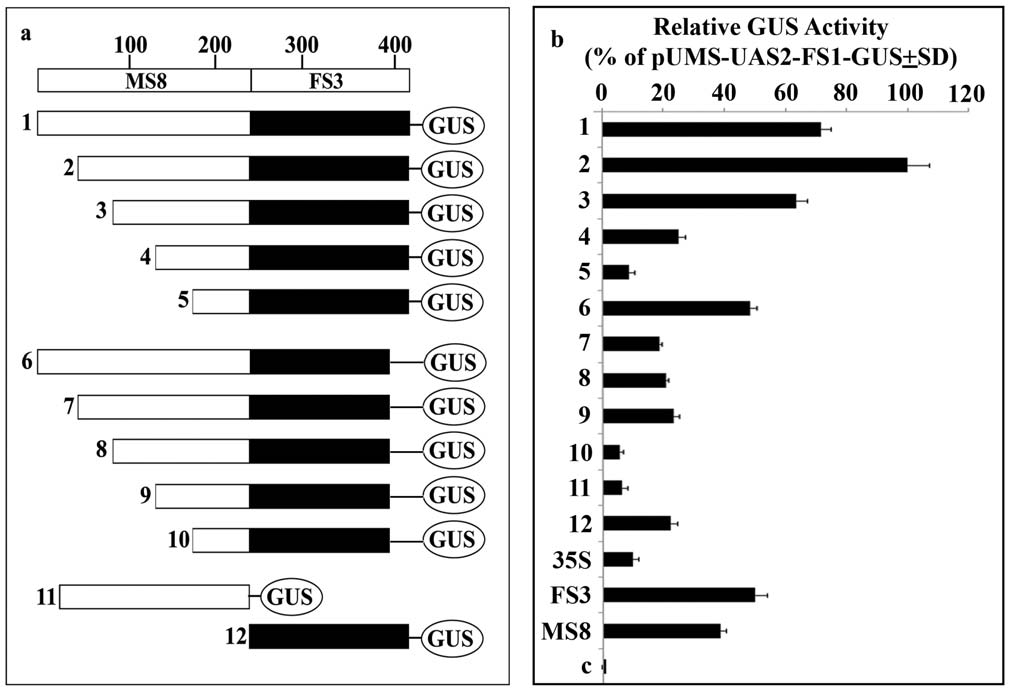
Transient assay of recombinant promoters in tobacco protoplasts. (a) A schematic map of the GUS constructs (number 1 to 12) developed for the recombinant hybrid promoter analysis, combining the fragments from the parental promoters MS8 (11) and FS3 (12) as indicated. At the top, relative size (bp) of the MS8 (open box) and FS3 (solid box) fragments and relative position of TATA box are shown. The coordinates of the respective domains of hybrid promoters were given in Table 1. (b) Transient expression analysis of MSgt-FSgt hybrid promoter in tobacco protoplasts using the GUS reporter gene. Five µg of soluble protein extract from transformed protoplasts were used for the GUS assay. The average GUS activity (as % of construct 2. pUMSgt-FSgtGUS ± SD) was presented in the histogram from five independent experiments for each construct assayed at least three times. Error bars show the 95% confidence intervals of the mean. Statistical (one-way analysis of variance, ANOVA) analysis showed an extremely significant P value of <0.05. Relative activities of parental promoter GUS construct (FS3 and MS8), pUCPMAGUS (CaMV35SGUS construct, 35S) and empty vector pUCPMA control (C), hybrid promoter GUS Constructs: 1, pUMS-UAS1-FS1GUS; 2, pUMS-UAS2-FS1GUS; 3, pUMS-UAS3-FS1GUS; 4, pUMS-UAS4-FS1GUS; 5, pUMS-UAS5-FS1GUS; 6, pUMS-UAS1-FS2GUS; 7, pUMS-UAS2-FS2GUS; 8, pUMS-UAS3-FS2GUS; 9, pUMS-UAS4-FS2GUS, 10, pUMS-UAS5-FS2GUS, 11, pUMS-UAS1GUS; 12, pUFS-1GUS were shown along with that obtained from FS3 (*Figwort mosaic virus* sub-genomic transcript promoter between coordinates −271 to +31) and MS8 (*Mirabilis mosaic virus* sub-genomic transcript promoter between coordinates −306 to +27) promoter individually.

Pararetrovirus-based recombinant sub-genomic transcript promoters (10) were constructed by combining five different upstream activation sequence (UAS): (MS-UAS1, MS-UAS2, MS-UAS3, MS-UAS4 and MS-UAS5) of the *Mirabilis mosaic virus* sub-genomic transcript promoter (MMVSgt) [22] with two different TATA box-containing promoter fragments (FS-1 and FS-2) of the *Figwort mosaic virus* sub-genomic transcript promoter (FS3) [23] (Figure 1). The boundaries of respective UAS (of MS8 promoter) and TATA-box-containing fragments (of FS3 promoter) present in each hybrid promoter were listed in Table 1. Different UASs of the MMV-Sgt promoter were PCR amplified individually using specific pairs of synthetic oligonucleotides (Table 2) containing the appropriate sequence to generate *Eco*RI and *Hin*cII sites at the 5′-end and *Sma*I and *Hind*III sites at the 3′-end. PCR-amplified fragments were digested with *Eco*RI and *Hind*III, gel-purified and cloned into the corresponding sites of the pUC119 vector. The resulting plasmids were designated as pUMS-UAS1, pUMS-UAS2, pUMS-UAS3, pUMS-UAS4 and pUMS-UAS5. Similarly, the FS-1 and FS-2 promoters were PCR-amplified using the FS3 promoter DNA as a template and synthetic primers (Table 2) containing the appropriate restriction sites to generate *Eco*RI and *Hin*cII overhangs at the 5′-end and *Sma*I and *Hin*dIII overhangs at the 3′-end. These fragments (5′-*Eco*RI – *Hinc*II- fragment – *Sma*I – *Hind*III-3′) were cloned into the corresponding sites of pUC119. These resulting plasmids were designated as pUFS-1 and pUFS-2. The 182-bp (FS-1) and 165-bp (FS-2) *Figwort mosaic virus* sub-genomic transcript promoter fragments were isolated as *Hin*cII – *Hin*dIII fragments from the pUFS-1 and pUFS-2 plasmids and then inserted individually into the pUMS-UAS1, pUMS-UAS2, pUMS-UAS3, pUMS-UAS4 and pUMS-UAS5 plasmids at the *Sma*I and *Hin*dIII sites. The resulting plasmids were designated as pUMS-UAS1-FS1, pUMS-UAS2-FS1, pUMS-UAS3-FS1, pUMS-UAS4-FS1, pUMS-UAS5-FS1, pUMS-UAS1-FS2, pUMS-UAS2-FS2, pUMS-UAS3-FS2, pUMS-UAS4-FS2 and pUMS-UAS5-FS2. All promoter inserts were subjected to nucleotide sequencing and the UAS portion of the MS8 promoter was linked to the TATA-containing promoter from FS3 via a *Sma*I site (cccggg) in all of the hybrid promoters. The hybrid promoter fragments, MS-UAS1-FS1 (419 bp), MS-UAS2-FS1 (369 bp), MS-UAS3-FS1 (319 bp), MS-UAS4-FS1 (269 bp), MS-UAS5-FS1 (219 bp), MS-UAS1-FS2 (402 bp), MS-UAS2-FS2 (352 bp), MS-UAS3-FS2 (302 bp), MS-UAS4-FS2 (252 bp) and MS-UAS5-FS2 (202 bp) were isolated by *Eco*RI and *Hind*III restriction digestion and sub-cloned into a plant protoplast expression vector containing the *GUS* reporter gene (pUCPMAGUS) by replacing the CaMV35S promoter [26]. The resulting plasmids were designated as pUPMS-UAS1-FS1GUS, pUPMS-UAS2-FS1GUS, pUPMS-UAS3-FS1GUS, pUPMS-UAS4-FS1GUS, pUPMS-UAS5-FS1GUS, pUPMS-UAS1-FS2GUS, pUPMS-UAS2-FS2GUS, pUPMS-UAS3-FS2GUS, pUPMS-UAS4-FS2GUS and pUPMS-UAS5-FS2GUS respectively.

**Table 1:** Upstream Activation Sequence (UAS) of MS8 and downstream TATA containing sequence of FS3 present in each recombinant promoter.

Sl. No.	Recombinant promoters	UAS sequence of MS8	Promoter sequence of FS3	Length of recombinant promoter
1	MS-UAS1-FS1	−356 to −125	−151 to +31	419 bp
2	MS-UAS2-FS1	−306 to −125	−151 to +31	369 bp
3	MS-UAS3-FS1	−256 to −125	−151 to +31	319 bp
4	MS-UAS4-FS1	−206 to −125	−151 to +31	269 bp
5	MS-UAS5-FS1	−156 to −125	−151 to +31	219 bp
6	MS-UAS1-FS2	−356 to −125	−151 to +14	402 bp
7	MS-UAS2-FS2	−306 to −125	−151 to +14	352 bp
8	MS-UAS3-FS2	−256 to −125	−151 to +14	302 bp
9	MS-UAS4-FS2	−206 to −125	−151 to +14	252 bp
10	MS-UAS5-FS2	−156 to −125	−151 to +14	202 bp

**Table 2:** List of oligonucleotide primers used for amplifying different promoter fragments and genes.

Name of PCR product	Forward primer sequence (5′–3′)	Reverse Primer sequence (5′–3′)
MS-UAS1 (−356 to −125)	actgaattcgtcgacagcggtaaaacaggtgattact	actaagcttcccgggtaattctctggtgagataatc
MS-UAS2 (−306 to −125)	actgaattcgtcgacgttttacagtcaggacagataat	actaagcttcccgggtaattctctggtgagataatc
MS-UAS3 (−256 to −125)	actgaattcgtcgacaaaaagattactggtgacagta	actaagcttcccgggtaattctctggtgagataatc
MS-UAS4 (−206 to −125)	actgaattcgtcgacgtggttttcacattacacctttaa	actaagcttcccgggtaattctctggtgagataatc
MS-UAS5 (−156 to −125)	actgaattcgtcgacatgtgctggctgattatctcacc	actaagcttcccgggtaattctctggtgagataatc
FS-1 (−151 to +31)	actgaattcgtcgactcgaacatcttgaaggtgtac	actaagcttcccgggcactccccctctctaaaaatt
FS-2 (−151 to +14)	actgaattcgtcgactcgaacatcttgaaggtgtac	actaagcttcccgggaaattttgtttttagaatttgtg
*GFP* *GAPDH*	actctcgagatgagtaaaggagaagaacttcagtaaacgacccgtaaatg	actgagctcttatttgtatagttcatccatggccagttggtgttaatgttt
*β-Actin* *GUS*	atgactcagatcatgtttgagactctcgagacaggcgattaaagagctgat	agccttcgcaatccacatctgactgagctccatgcatacaccactttgcta

### Construction of MSgt-FSgt (MS-UAS2-FS1), FS3, MS8, MS-UAS2, FS-1 and CaMV35S promoter-GUS vectors for transient assay

The hybrid promoter MS-UAS2-FS1 and the respective clone (constructed as above) are hereinafter referred to as the MSgt-FSgt hybrid promoter and pUPMSgt-FSgt, respectively. The boundaries of the MS8 and FS3 promoter fragments present in the MSgt-FSgt promoter (MS-UAS2-FS1) are shown in Table 1.

The upstream activation sequence (-306 to -125) of the MMV-Sgt promoter (MS-UAS2) and the TATA-containing sequence (−151 to +31) of the FMV-Sgt promoter (FS-1) were cloned into *Eco*RI and *Hind*III sites of pUCPMAGUS vector [26]. The resulting plasmids were designated as pUPMS-UAS2GUS and pUPFS-1GUS.

### Promoter-GFP constructs for transient assay

The *GFP* cDNA gene was PCR-amplified using a synthetic primer pair (Table 2) to insert the *Xho*I site at the 5′-end and the *Sst*I site at the 3′-end. The amplified *GFP* gene was digested with *Xho*I and *Sst*I and subsequently cloned into the *Xho*I and *Sst*I sites of pUCPMAGUS, pFS3GUS, pMS8GUS and pUPMSgt-FSgtGUS (set-a) replacing the *GUS* gene to generate plasmids pUCPMAGFP, pFS3GFP, pMS8GFP and pUPMSgt-FSgtGFP (set-b) respectively.

### Construction of plant expression vectors

The MSgt-FSgt promoter fragment 369-bp from pUPMSgt-FSgt was gel purified (as *Eco*RI – *Hind*III fragment) and inserted into pKYLXGUS, a plant expression vector [26], [27] using *Eco*RI and *Hin*dIII sites that flank the promoter, thereby replacing the CaMV35S promoter. The resulting plant expression vector was designated as pKMSgt-FSgtGUS.

### Protoplast isolation, electroporation and transient assay of recombinant promoters

Protoplasts from a tobacco cell suspension culture (*Nicotiana tabacum* cv. Xanthi Brad) were purified on a 20% sucrose gradient after digestion by cellulase (Sigma, USA) and pectinase (Sigma, USA) and electroporated following a standard protocol as previously described [26]. In brief, an aliquot of 750 µl containing 2×10^6^ protoplasts were electroporated (200 V used for charging 965-µF capacitance for 40–50 ms) with 5 µg of each of the following promoter constructs: pUPMS-UAS1-FS1GUS, pUPMS-UAS2-FS1GUS, pUPMS-UAS3-FS1GUS, pUPMS-UAS4-FS1GUS, pUPMS-UAS5-FS1GUS, pUPMS-UAS1-FS2GUS, pUPMS-UAS2-FS2GUS, pUPMS-UAS3-FS2GUS, pUPMS-UAS4-FS2GUS and pUPMS-UAS5-FS2GUS using an electroporation cuvette (0.4-cm electrode gap). The plasmid pUCPMAGUS containing the CaMV35S promoter [26] was used as a control to compare the activities of the promoters.

GUS activities in transformed protoplasts were measured after 20 h of incubation at 28°C [28]. The average activities of these promoter constructs were expressed as the mean of five successive independent experiments.

### Promoter expression analysis using CLSM

Tobacco protoplasts were electroporated with promoter constructs carrying *GUS* reporter (set a) and *GFP* reporter (set b) constructs for assaying their transient activities as described earlier. Protoplasts electroporated with promoter constructs carrying the *GUS* reporter (set-a) were incubated in a 1mM MUG (4-methyl-umbelliferyl-beta-d-glucuronide) at 37°C for 30 in to generate 4-MU (7-hydroxy-4-methylcoumarin) for the detection of GUS localization (as blue fluorescence) in protoplasts using CLSM (TCS SP5, Leica Microsystems CMS GmbH, D-68165 Mannheim, Germany) with similar specifications as described earlier [29]. Similarly, to excite the expressed GFP [30], [31] in protoplasts, the argon laser (40%) with AOTF of 488 nm (at 30%) was used. The fluorescence emissions were collected between 501 and 598 nm as described earlier [29]. Following image acquisition, 4-MU (for GUS) and GFP fluorescence intensities were quantified using the LAS AF Software as per the instructions of Leica Microsystems. The GFP and GUS (4-MU) fluorescence intensities from 200 individual protoplasts were assayed, and the mean data were presented with respective ± SD.

### Tobacco plant transformation and analysis of transgenic plants

The following constructs, pKYLX (vector) [27], pKYLXGUS (containing the CaMV35S promoter), pKMS8GUS [22], pKFS3GUS [23], and pKMSgt-FSgtGUS were introduced into the *Agrobacterium tumefaciens* strain C58C1:pGV3850 by the freeze thaw method [32]. Tobacco plants (*Nicotiana tabacum* cv Samsun NN) were transformed with the engineered *Agrobacterium* as previously described [33]. Ten to twelve (10–12) independent plant lines were generated for each construct and maintained under green house conditions (photoperiod: 16/8 hour at 220 µmole m^−2^ s^−1^, Temperature: 28°±3°C, Humidity: 70–75%). Kanamycin-resistant plants (T_1_ generation) were used for further analysis. GUS activity in seedling, root, leaf and stem were measured according to the protocol described earlier [28], [34]. Transgenic seedlings obtained from each construct were subjected to histochemical GUS staining using 1% X-gluc solution.

### Transformation of *Arabidopsis thaliana* plants and analysis of transgenic plants

*Arabidopsis thaliana* (ecotype Columbia) plants were transformed by pKYLXGUS and pKMSgt-FSgtGUS by floral dip method [35]. The Arabidopsis flower buds were dipped in an Agrobacterium cell suspension containing freshly prepared 5% sucrose (wt/vol) and 0.02–0.05% Silwet L-77 (vol/vol) for 30–45 sec. After that these plants were grown at 22°C under long day condition (16 hours light and 8 hours dark cycle) till setting of seeds. Seeds were collected after maturation and dried. After surface sterilization, seeds were suspended in sterile 0.05% agarose and spreaded on MS selection plate (4.3 g Murashige & Skoog salts, 10 g sucrose, 0.5 g MES, 8 g agar per liter; pH 5.7, Kanamycin 100 mg/l and Cefotaxime 100 mg/l) and allowed to germinate. Only true transformants produced green healthy leaves (non-transformants became dried and bleached). A total number of 75 independent transgenic plants were raised. GUS activities in whole seedlings (21 days old) were measured according to the protocol described earlier [28]. Histochemical GUS staining was performed as described earlier.

### Molecular analysis of transgenic plants: RNA isolation, northern blot analysis

Total RNA was obtained from transgenic seedlings expressing pKYLX, pKYLXGUS, pKMS8GUS, pKFS3GUS and pKMSgt-FSgtGUS using the RNeasy plant mini Kit (Qiagen, Tokyo, Japan) according to manufacturer's protocol. The *GUS* and β-*Actin* genes were PCR-amplified from pKYLXGUS plasmid (for *GUS*) and tobacco genomic DNA [36] respectively. The PCR fragments were gel-purified using the QIAquick gel extraction kit (Qiagen, Tokyo, Japan) and was labeled with [α- P^32^] dCTP using the Random Primer labeling Kit (NEBlot Kit, New England Biolabs Inc., MA, USA). Total RNA (5 µg) isolated from the transgenic seedlings was subjected to electrophoresis in a 1.2% agarose gel containing 0.66 M formaldehyde and then transferred overnight to an IMMOBILON-NY+ membrane (Millipore, Billerica, MA, USA) by capillary action in 10X SSC (1.5M NaCl, 150 mM sodium citrate). Hybridization with the ^32^P labeled *GUS*-probe was carried out at 65°C overnight and northern blot analysis was carried out using standard protocol [37]. The washed membrane with the bound probe was scanned with the FLA-5000 Imaging System (Fuji FILM Life Science, USA). The membrane was subsequently washed at 80°C in a solution containing 1X SSC, 0.1% SDS, and 0.05% Na–pyrophosphate to remove the *GUS* probe. The stripped membrane was then re-probed with the β-*Actin* to ensure equal loading.

### Real-time PCR analysis of *GUS* transcripts driven by MS8, FS3, CaMV35S and MSgt-FSgt promoter

Total RNA was isolated from transgenic seedlings expressing the pKMS8GUS, pKFS3GUS, pKYLXGUS, and pKMSgt-FSgtGUS promoter constructs and subsequently mRNA was isolated from total RNA using the PolyATract mRNA isolation system (Promega, Madison, WI, USA). About 2.0 µg of mRNA was used to synthesize the first strand cDNA using AMV reverse transcriptase and oligo (dT) primers at 42°C for 1.5 hours. Real time PCR reactions were performed using SYBR Premix Ex TaqTM II (Perfect Real Time, Takara Bio Inc.) according to manufacturer's instruction on an Opticon-2 qRT-PCR instrument (MJ Research, Bio-Rad; Model CFD-3220). *GAPDH* cDNA was used as an internal control for normalization of *GUS* mRNA levels in the real time PCR. The PCR cycling program was conducted as follows; 94°C for 30 sec followed by 37 cycles of 94°C for 5 sec, 57°C for 30 sec, 72°C for 30 sec and finally 72°C for 5 minutes. The GUS mRNA levels in MS8, FS3 and MSgt-FSgt and CaMV35S promoters were calculated using the 2^−ΔΔCT^ method [38], [39] and data presented here are relative to CaMV35S promoter line.

### Quantitative reverse transcription-polymerase chain reaction (qRT-PCR)

First strand cDNA was synthesized using 2.0 µg mRNA purified from total RNA extracted from transgenic tobacco seedlings transformed with pKYLX, pKYLXGUS, pKMS8GUS, pKFS3GUS and pKMSgt-FSgtGUS and transgenic Arabidopsis seedlings transformed with pKYLX, pKYLXGUS and pKMSgt-FSgtGUS as described above. PCR amplifications of *GUS* and *GAPDH* were performed using 1 µl of first strand reaction products in the presence of gene specific primers (Table 2). Twenty-six cycles of PCR was conducted with denaturation at 92°C for 1 minute, annealing at 60°C for 30 seconds and extension at 72°C for 1 minute. Eight µl samples of each PCR reaction were analyzed on 1% agarose gel.

### Preparation of transgenic plant samples for CLSM analysis

Transgenic tobacco plants were generated using the MSgt-FSgt and CaMV35S promoters fused to the *GUS* reporter gene as described earlier. The transgenic plant parts were initially kept under vacuum infiltration for 10 min and then incubated at 37°C overnight in the presence of 1 mM MUG to produce 4-MU. The transverse sections (approximately 80 micron thick) of leaf blade, leaf midrib, stem and root were obtained by sectioning the treated plant tissue using a microtome (Cryostat, Leica CM 1850). Fluorescence images of thin transverse sections of these tissues were captured using a CLSM. To quantify GUS activity, the treated tissue sections were excited with the 405 diode laser (Argon 364 nm UV laser may be more appropriate), and fluorescence emissions were collected between 440 and 455 nm with the detector (PMT) gain set at 1150V. Intracellular GUS localizations were detected by blue fluorescence of 4-MU using CLSM and intensities of blue-fluorescence were measured from different cells according to the protocol described earlier [29].

### Nuclear protein binding assay for the hybrid promoter

Tobacco nuclear extracts were prepared from greenhouse-grown leaves using standard protocol [40] with slight modifications. The MSgt-FSgt promoter DNA fragment (369 bp) was labeled using Prime-a-gene labeling system (Promega, USA) in the presence of γ-P^32^ dATP at 37°C for 1 hr. The EMSA binding reaction was carried out in a 30-µl volume containing ^32^P-labeled MSgt-FSgt probe in binding buffer (100 mM Tris-HCl pH 7.5, 0.5 mM DTT, 1 mM EDTA, 7% v/v glycerol, 1 mM PMSF, 5 µg/µl BSA, 0.1–0.2 µg/µl poly dI-dC, 40–50 mM NaCl) in the presence of nuclear protein extract (5–6 µg). Reactions were incubated at room temperature for 30 min. The DNA-protein complexes were resolved in non-denaturing 5% polyacrylamide gel [40]. Competitive EMSA reactions were carried out in the presence of the ^32^P-labeled MSgt-FSgt DNA (as probe) and 20, 50 and 100 fold excesses of non-labeled DNA (MSgt-FSgt).

DNaseI foot-printing experiments were carried using standard protocol [41]. The probe (end-labeled MSgt-FSgt promoter) was incubated in the presence of 100 mM Tris-HCl pH 7.5, 0.5 mM DTT, 1 mM EDTA, 7% v/v glycerol, 1 mM PMSF, 5 µg/µl BSA and 0.1–0.2 µg/µl polydI-dC for 30 min at room temperature. DNaseI (0.025 U/µl) was added and incubated for 45 sec at room temperature with 5 µg and 10 µg of nuclear protein separately. The reaction products were then denatured and loaded onto a 6% denaturing polyacrylamide sequencing gel along with sequencing reactions for the MSgt-FSgt DNA fragment (Thermo Sequenase Cycle Sequencing Kit; USB Corporation, Cleveland, OH, USA).

### *In vitro* methylation assay

The putative CG methylation sensitive sites present in MSgt-FSgt and the CaMV35S promoters were determined *in-silico* using Support Vector Machine (http://bio.dfci.harvard.edu/Methylator) [42]. The effect of in vitro methylation on the transient expression of MSgt-FSgt and CaMV35S promoters coupled to GUS reporter gene were investigated according to Pradhan et al. [43] with some modifications. Plasmids pUPMSgt-FSgtGUS and pUCPMAGUS individually were grown in a dam^−^ and dcm^−^ E.coli K12 strain ER2925 and purified. DNA methyltransferase *M.SssI* was used to methylate each plasmid construct at 37°C for 1.5 hours in presence of 0.5 mM S-adenosyl methionine. The methylated plasmids were purified and quantified using a spectrophotometer (CECIL BioQuest CE 2501 CECIL Instruments Ltd, England). Approximately 5 µg each methylated and unmethylated plasmids from both constructs were used for transient assay in a protoplast system (*Nicotiana tabaccum* cv. Xanthi brad) following protocol as described earlier.

### Statistical Analysis

Statistical analyses were performed by using Graph Pad Prism (version 5.01). Unpaired students *t* test was used for analyzing the activity of methylated-MSgt-FSgt and methylated-CaMV35S promoters. A *P* value of <0.05 was considered to reveal a significant difference.

## Results

### Comparison of activity of recombinant promoters with MS-UAS2, FS-1, FS3, MS8 and CaMV35S promoters in transient protoplast assay

Recombinant promoters (Figure 1a, Table 1) fused to the *GUS* reporter gene were tested individually for their transcriptional activities using a transient tobacco protoplast assay (*Nicotiana tabacum* cv. Xanthi Brad). Transformed protoplast with empty vector (pUCPMA) was used as a control. As shown in Figure 1b, the relative GUS activities of all hybrid promoters were expressed considering the activity of MSgt-FSgt promoter as 100%. The GUS activities obtained from the hybrid constructs MS-UAS1-FS1, MSgt-FSgt, MS-UAS3-FS1, MS-UAS4-FS1, MS-UAS1-FS2 and MS-UAS4-FS2 were found to be 7.32, 10.31, 6.49, 2.54, 4.93 and 2.37 times stronger, respectively, than the CaMV35S promoter (Figure 1b). The recombinant promoter, MSgt-FSgt, showed maximum GUS activity, approximately10 fold higher than that of CaMV35S promoter. The activity of MSgt-FSgt was found to be 5.22, 13.99, 2.18 and 2.9 times stronger than the FS-1, MS-UAS-2, FS3 [23] and MS8 [22] promoters respectively (Figure 1b).

### Comparison of promoter activities of MS8, FS3, CaMV35S and MSgt-FSgt fused to GUS and GFP reporters using CLSM

**Figure 2:**
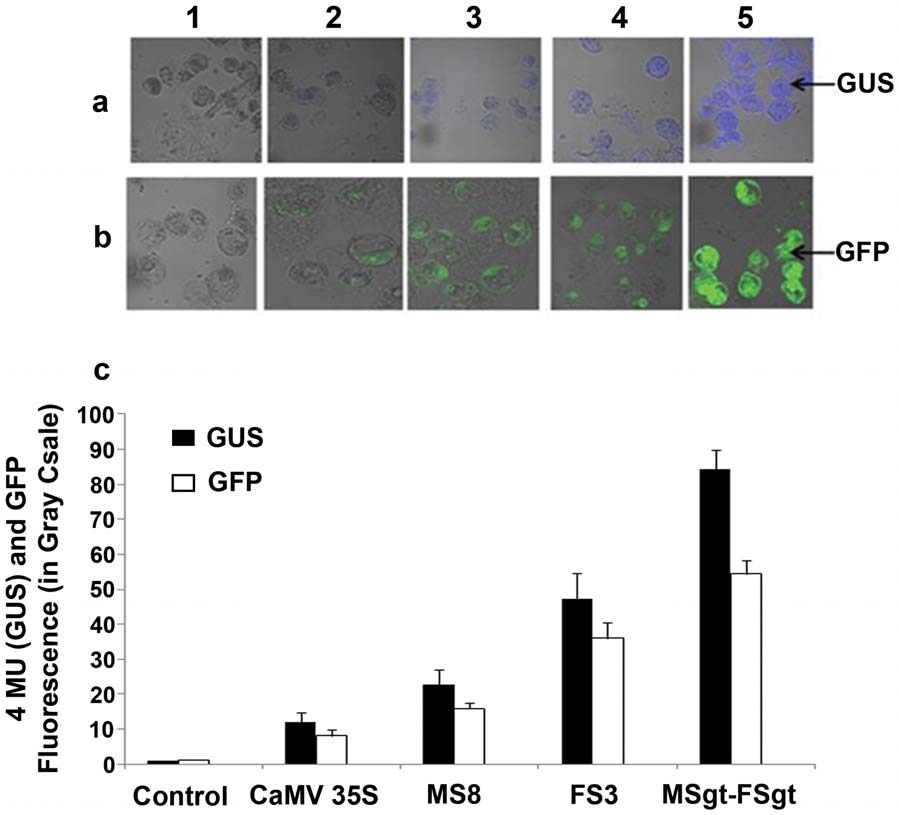
Fluorescence detection and transient assay of GUS (as 4-MU fluorescence) and GFP using a confocal laser scanning microscope (CLSM) for parental and hybrid promoters in tobacco protoplasts. (a) GUS activity as 4-MU fluorescence recorded in confocal microscope as described in methods for the promoter-GUS constructs: 1, pUCPMA (empty vector control, with no GUS); 2, pUCPMAGUS (with CaMV35S promoter) 3, pMS8GUS (with MS8 promoter [22]); 4, pFS3GUS (with FS3 promoter [23]) and 5, pUPMSgt-FSgtGUS (recombinant promoter). (b) GFP fluorescence recorded in confocal microscope as described in methods for the promoter-GFP constructs: 1, pUCPMA (empty vector control, with no GFP); 2, pUCPMAGFP (with CaMV35S promoter); 3, pMS8GFP (with MS8 promoter); 4, pFS3GFP (with FS3 promoter); and 5, pUPMSgt-FSgtGFP (hybrid promoter). (c) Transient assay of 4-MU fluorescence (for GUS) and GFP using CLSM for parental and hybrid promoters in tobacco protoplasts, fluorescence intensities for GUS and GFP were measured as described in methods and presented in a bar diagram as average value ± SD of two independent experiments, each performed in triplicate, for the GUS and GFP constructs as described in panel a and b: empty vector with no GUS or GFP (Control), CaMV35S, MS8, FS3 and MSgt-FSgt promoter constructs. (CaMV35S: *Cauliflower mosaic virus*; MS8: *Mirabilis mosaic virus* sub-genomic transcript promoter; FS3: *Figwort mosaic virus* sub-genomic transcript promoter; MSgt-FSgt: the hybrid promoter).

Tobacco protoplasts were transformed with two different sets of promoter constructs as set-a and set-b separately as described in methods. In both cases the pUCPMA vector was used as a control. Blue fluorescence of transformed protoplasts obtained from using set-a (with *GUS* reporter gene; Figure 2a) were captured by CLSM as described in the Method section, and were presented. Similarly, the GFP fluorescence images of protoplasts transformed with set-b constructs were presented in Figure 2b. The intensity of the blue fluorescence due to 4-MU (for GUS) and green fluorescence (for GFP) from the individual promoter construct was quantified from 200 independent transformed protoplasts using the LAS-AF software. CLSM-based analysis of the GUS reporter (blue fluorescence) demonstrated that the activity of the MSgt-FSgt promoter was 1.79, 3.73 and 6.96 times stronger than that of the FS3, MS8 and CaMV35S promoters, respectively (Figure 2c). The expression level of the *GFP* reporter gene under the control of these promoters as measured by CLSM revealed that the MSgt-FSgt promoter was 1.51, 3.43 and 6.81 times stronger than the FS3, MS8 and CaMV35S promoters (Figure 2c).

### Analysis of transgenic tobacco plants developed with MS8, FS3, CaMV35S and MSgt-FSgt promoter constructs

**Figure 3:**
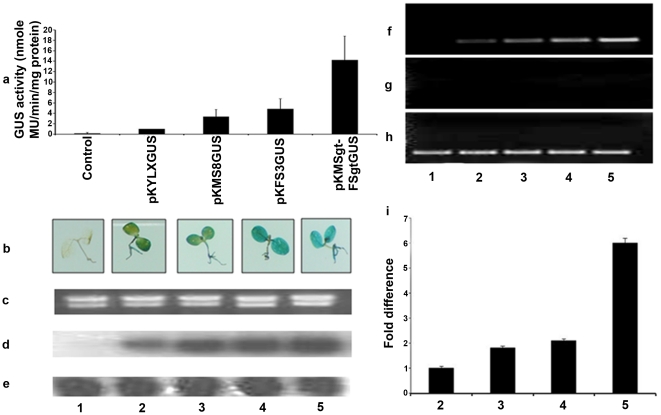
Expression analysis of parental and recombinant hybrid promoters in transgenic tobacco plants. (a) Promoter activity was monitored in 21-day old tobacco (*N. tabacum* cv Samsun NN) seedlings (R1 progeny, 2nd generation, Kan^R^) grown aseptically on an MS-agar medium in presence of kanamycin (200 µg/ml) and 1% sucrose. Soluble protein extracts (5 µg) from whole seedlings were used for the GUS assay. The data present average ±SD of four independent experiments for each construct: pKYLX (empty vector control), pKYLXGUS (with the CaMV35S promoter), pKMS8GUS (with MS8 promoter), pKFS3GUS (with FS3 promoter), pKMSgt-FSgtGUS (hybrid promoter). (b) Histochemical localization of GUS activity (blue coloration) in transgenic tobacco seedlings (magnification x 10.0) obtained from control (1), CaMV35S (2), MS8 (3) FS3 (4), and MSgt-FSgt (5) promoter constructs represents the relative strength of the promoter constructs. (c) Display of electrophoresis of total RNA, obtained from 21 days old tobacco seedlings developed with control (1), CaMV35S (2), MS8(3), FS3 (4), and MSgt-FSgt (5) promoter constructs used as loading control. (d) Northern blot detection of the GUS reporter gene in transgenic tobacco developed with control (1), CaMV35S (2), MS8 (3), FS3 (4) and MSgt-FSgt (5) promoter constructs. (e) The same membrane was re-probed with ^32^P-labelled *β-Actin* gene to confirm the equal loading of RNA samples. (f) Electrophoresis of RT-dependent PCR amplifications of GUS transcripts from total RNA obtained from transgenic plant generated for empty vector control (1), CaMV35S (2), MS8 (3), FS3 (4), and MSgt-FSgt (5) promoter constructs as described in (a). (g) Electrophoresis of RT-independent (-RT) PCR amplifications of GUS transcripts from total RNA obtained from transgenic plant developed for empty vector control (1), CaMV35S (2), MS8 (3) FS3 (4), and MSgt-FSgt (5) promoter constructs. Electrophoresis was performed to check the presence of DNA in RNA preparation. (h) Electrophoresis of RT-dependent PCR amplifications of GAPDH transcripts from total RNA from transgenic plant developed for empty vector control (1), CaMV35S (2), MS8 (3), FS3 (4), and MSgt-FSgt (5) promoter constructs. (i) The relative abundance of GUS transcript (data present average fold differences of GUS transcript ± SD of two independent experiments) assayed by real-time quantitative reverse transcription PCR (RT-qPCR). Total RNA was isolated from transgenic seedlings (2^nd^ generation, 21-day old) developed for pKYLX (empty vector control), pKYLXGUS (with CaMV35S promoter), pKMS8GUS (with MS8 promoter), pKFS3GUS (with FS3 promoter), pKMSgt-FSgtGUS (hybrid promoter).

Transgenic tobacco plants were generated using the promoter constructs pKYLX (empty vector), pKYLXGUS, pKMS8GUS, pKFS3GUS, pKMSgt-FSgtGUS. No phenotypic changes were marked between control and transgenic tobacco plants expressing these promoter constructs. Total proteins isolated from T_1_ seedlings were used to assess GUS activity [28]. A comparison of the GUS activities of the various constructs with the CaMV35S (considered as 1) was presented in Figure 3a. In transgenic plants, the MSgt-FSgt promoter was found to exhibit 14.22, 4.26 and 2.94 times stronger activity, compared to the CaMV35S, MS8, and FS3 promoters, respectively (Figure 3a).

Histochemical staining of transgenic tobacco seedlings (T_1_ generation, 21 days old) generated with the CaMV35S, MS8, FS3 and MSgt-FSgt promoter constructs were presented in Figure 3b. When GUS activities were measured from soluble proteins extracted from the roots, leaves and stems of transgenic seedlings, the relative GUS activities in the tissues were in the following order: roots> leaves> stems (data not shown).

Northern blot analysis was carried out using total RNA isolated from transgenic seedlings generated with pKYLX, pKYLXGUS, pKMS8GUS, pKFS3GUS, pKMSgt-FSgtGUS. Equal loading of RNA for the northern blot analysis was confirmed (Figure 3c) and the results were shown in Figure 3d. The highest intensities of GUS transcript accumulation were observed for the MSgt-FSgt promoter, followed by the FS3, MS8 and CaMV35S promoters. The membrane was washed and reprobed using the β-*Actin* probe as discussed in the Methods for further confirmation of equal loading as shown in Figure 3e.

Accumulation of *GUS*-specific mRNAs driven by each promoter in transgenic tobacco plants (T_1_ generation) were determined by semi-quantitative RT-PCR and real time PCR. The RT-dependent PCR amplifications of *GUS* transcript driven by the CaMV35S, MS8, FS3 and MSgt-FSgt promoters in transgenic plants were shown in Figure 3f. Accumulation of GUS transcripts was found to be the highest in transgenic plants carrying the MSgt-FSgt promoter followed by the FS3, MS8 and CaMV35S promoters. The fold differences in the *uid*A-mRNA expression levels for CaMV35S, MS8, FS3 and MSgt-FSgt promoter constructs were measured by real-time PCR. Results were presented as the mean of three independent experiments with respective standard deviation (± SD) in Figure 3i.

### Comparison of MSgt-FSgt and CaMV35S promoter activity in transgenic *Arabidopsis* plant

We have generated approximately 75 independent transgenic *Arabidopsis* plants expressing following promoter constructs pKYLX (empty vector), pKYLXGUS, pKMSgt-FSgtGUS individually as described in Methods. Furthermore, transgenic Arabidopsis plants expressing MSgt-FSgt promoter were found to be phonotypically identical with wild type plants.

**Figure 4:**
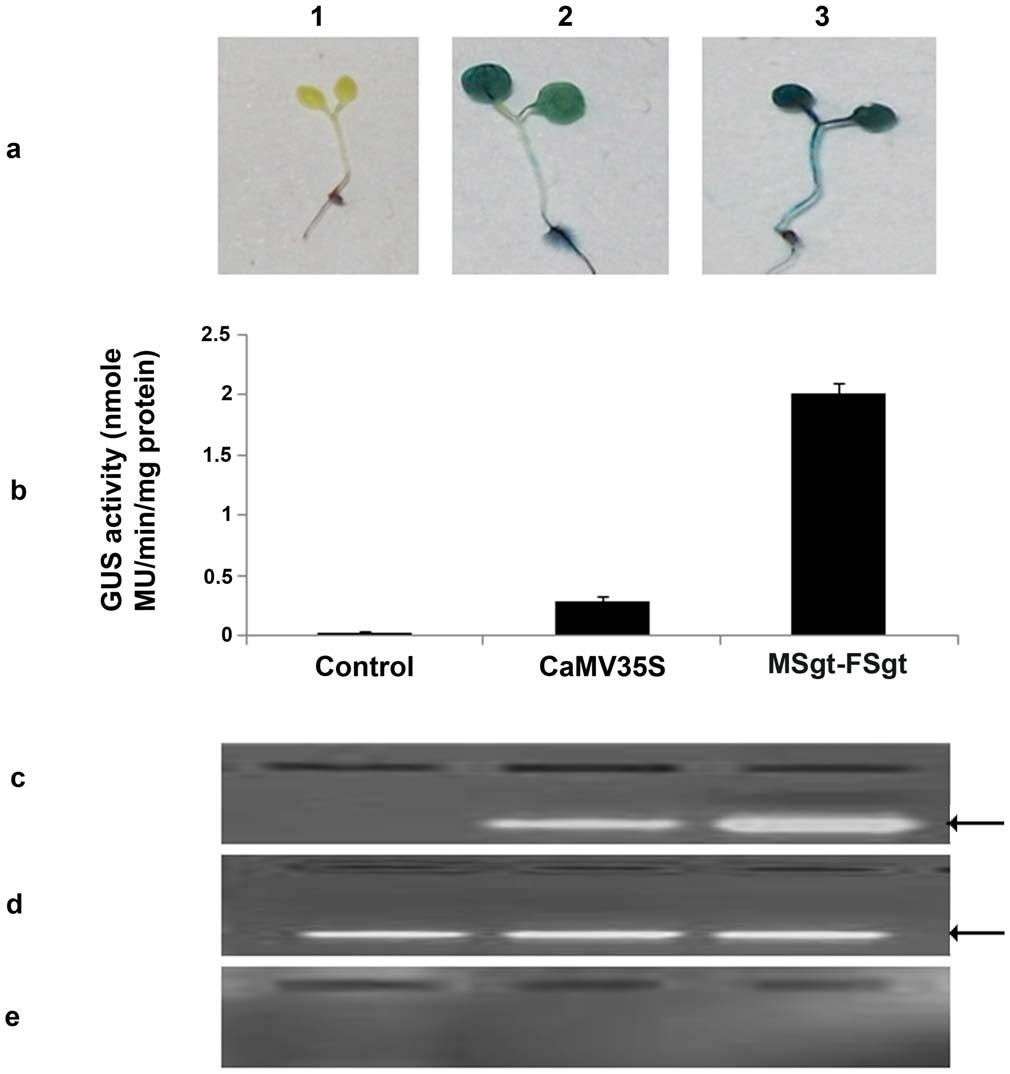
Comparative expression analysis of CaMV35S promoter and recombinant hybrid promoter in transgenic *Arabidopsis* plants. (a) Histochemical localization of GUS activity (blue coloration) in 21 days old transgenic *Arabidopsis* seedlings (magnification x 10.0) developed for the constructs: pKYLX (empty vector, Control), pKYLXGUS (with the CaMV35S promoter, 35S), and pKMSgt-FSgtGUS (hybrid promoter, MSgt-FSgt). (b) Promoter activity was assayed in 21 days old Arabidopsis seedlings (R1 progeny, 2nd generation, Kan^R^) grown aseptically on an MS-agar medium in presence of kanamycin (100 µg/ml) and 1% sucrose. Soluble protein extracts (5 µg) from whole seedlings were used for the GUS assay. The data present average ± SD of four independent experiments for each construct: pKYLX (empty vector control), pKYLXGUS (with the CaMV35S promoter), and pKMSgt-FSgtGUS (hybrid promoter); plasmids construction strategy described in the methods section. (c) Electrophoresis of RT-dependent PCR amplifications of GUS transcripts from total RNA isolated from transgenic *Arabidopsis* plant developed for pKYLX (empty vector control), pKYLXGUS (with CaMV35S promoter), and pKMSgt-FSgtGUS (hybrid promoter); plasmids construction strategy described in the methods section, the arrow indicating expected band. (d) Electrophoresis of RT-dependent PCR amplifications of GAPDH transcripts from total RNA obtained from transgenic plant developed for empty vector (1), CaMV35S (2), and MSgt-FSgt (3) promoter constructs, arrow indicating expected band. (e) Electrophoresis of RT-independent (-RT) PCR amplifications of GUS transcripts from total RNA obtained from transgenic plant developed for empty vector (1), CaMV35S (2), and MSgt-FSgt (3) promoter constructs showing no amplification (to check the presence of genomic DNA in the RNA preparation).

Histochemical staining of transgenic *Arabidopsis* seedlings expressing MSgt-FSgtGUS and CaMV35SGUS constructs were shown in Figure 4a; and the corresponding GUS activities in Figure 4b. The GUS activity of MSgt-FSgt was 7.16 times stronger than the CaMV35S promoter activity (Figure 4b). In the transgenic Arabidopsis plant, the RT-dependent PCR (semi q-RT PCR) amplification of *GUS* transcripts driven by the CaMV35S and MSgt-FSgt promoters were determined and it was observed that accumulation of GUS transcripts was more in transgenic Arabidopsis plants harboring the MSgt-FSgt promoter compared to that in transgenic plants with the CaMV35S promoter (Figure 4c).

**Figure 5:**
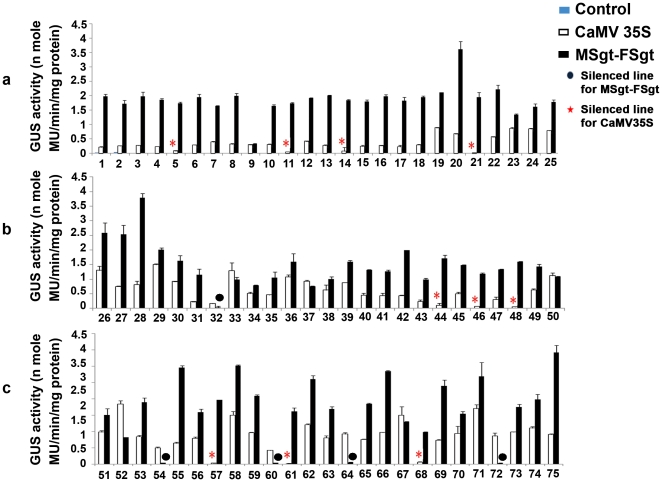
Expression analysis of CaMV35S and MSgt-FSgt promoters in transgenic *Arabidopsis* plants. Seventy five independent transgenic *Arabidopsis* lines were generated with the constructs pKYLXGUS and pKMSgt-FSgtGUS as described in methods. The average GUS activity ± SD from two independent experiments of 75 independent lines (21 days old) developed for each construct pKYLXGUS (35S-GUS), pKMSgt-FSgtGUS (MSgt-FSgtGUS) and empty vector pKYLX (control) are presented in the panel (a) line number 1 to 25, panel (b) line number 26 to 50 and panel (c) line number 51 to 75. Silenced (very low/basal level expression) lines were shown as different symbols for the CaMV35S and MSgt-FSgt promoters.

The average GUS activity of 75 independent lines (21 days old) from each construct was evaluated from two independent experiments and the results were presented in Figure 5. Approximately 10 out of 75 (13.3%) independent transgenic *Arabidopsis* lines expressing CaMV35S showed the basal or minimum level of GUS expression while in case of MSgt-FSgt promoter 5 lines out of 75 (6.7%) transgenic *Arabidopsis* lines showed the basal or minimum level of GUS expression (Figure 5a, 5b and 5c).

### Cell/Tissue specificity of MSgt-FSgt and CaMV35S promoter

**Figure 6:**
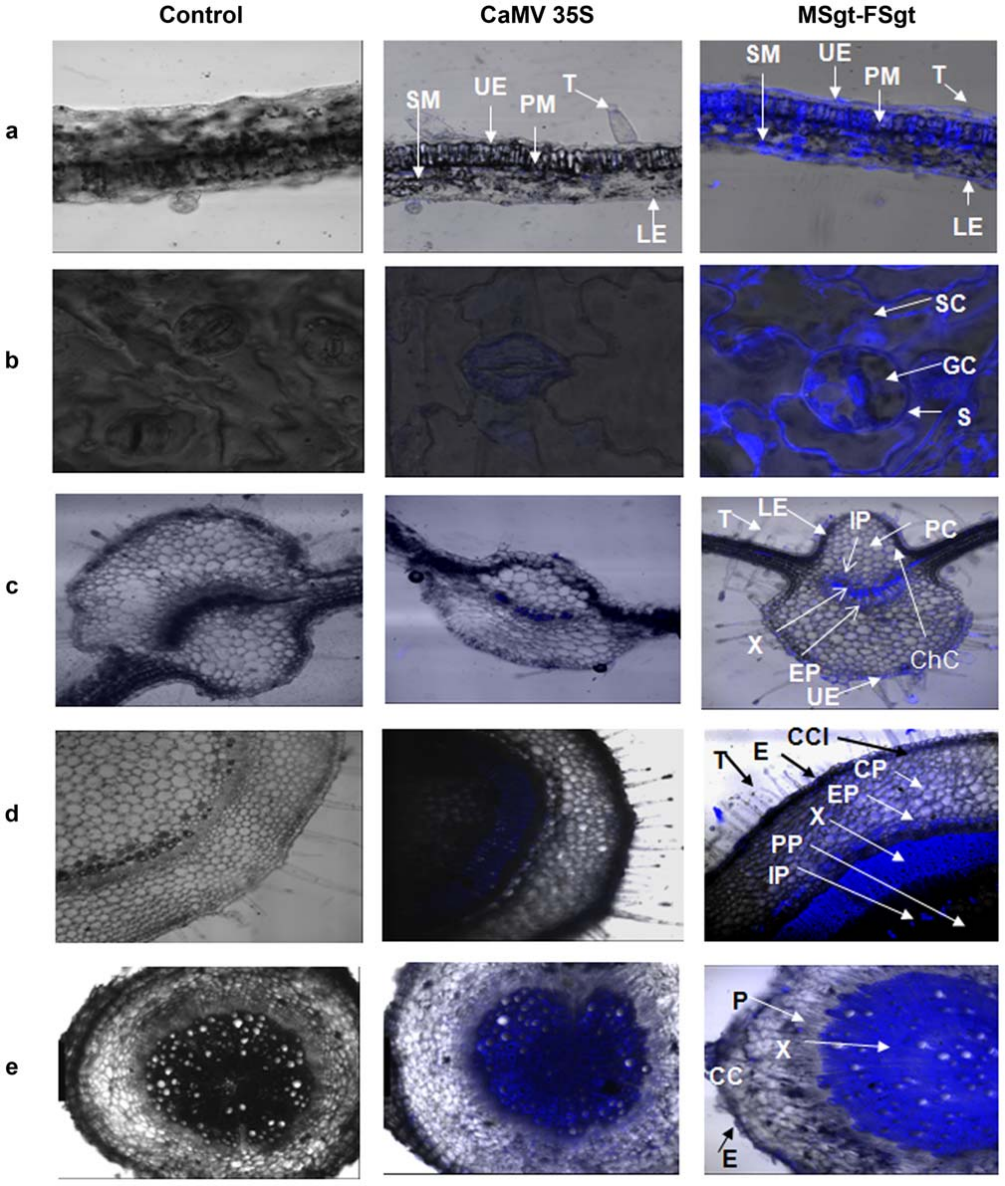
Tissue specific expression of the GUS reporter gene directed by either CaMV35S or MSgt-FSgt promoter in transgenic tobacco. (a) The superimposed images of transmitted and blue fluorescence of transverse sections of leaf blade from wild plants (*N. tabacum* cv. Samsun NN, as control) and transgenic plants for CaMV35S and MSgt-FSgt promoters are presented. SM: Spongy mesophyll; UE: Upper epidermis; PM: Palisade mesophyll; T: Trichomes; and LE: Lower Epidermis. (b) The superimposed image of transmitted and blue fluorescence of few representative S: stomata from the leaf blade of wild plants (as control) and transgenic plants raised for CaMV35S and MSgt-FSgt promoter are presented. SC: Subsidiary cells; GC: Guard cells. (c) The superimposed image of transmitted and blue fluorescence of transverse sections of the leaf midrib from wild type plants (as control) and transgenic plants raised for CaMV35S and MSgt-FSgt promoters are presented. UE: Upper epidermis; T: Trichomes; LE: Lower Epidermis; ChC: Collenchymatous cells; PC: Parenchymatous cells; IP: Internal Phloem; X: Xylem; EP: External Phloem. (d) The superimposed image of transmitted and blue fluorescence of transverse sections of the stem from wild type plants (as control) and transgenic plants raised by CaMV35S and MSgt-FSgt promoters are presented. T: Trichomes; CCl: Cortical collenchyma; PP: Pith parenchyma; CP: Cortical parenchyma; E: Epidermal cells; IP: Internal Phloem; X: Xylem; EP: External Phloem. (E) The superimposed image of transmitted and blue fluorescence of transverse sections of root from wild type plants (as control) and transgenic plants developed for CaMV35S and MSgt-FSgt promoter is presented. E: Epidermal cells; CC: Cortical cell; X: Xylem; P: Phloem.

**Figure 7:**
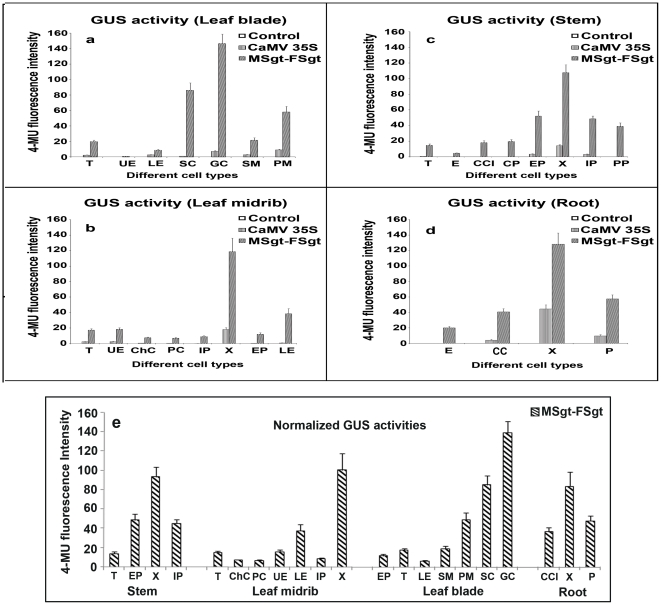
Expression of CaMV35S promoter and MSgt-FSgt promoter in different cell/tissue type of transgenic tobacco (*N. tabaccum* cv Samsun NN) lines assayed in confocal laser scanning microscope (CLSM). (a) The bar diagram denotes GUS activities (4- MU Fluorescence) of the different cell types of the leaf blade from wild type plant (*N. tabaccum* cv Samsun NN, as untransformed control), and transgenic plants generated for the GUS construct: pKYLXGUS (with CaMV35S promoter) and pKMSgt-FSgtGUS (with hybrid promoter). Different cell/tissue type of leaf blade is presented. T: Trichomes; UE: Upper epidermis; LE: Lower Epidermis; SC: Subsidiary cells; GC: Guard cells; SM: Spongy mesophyll; PM; Palisade mesophyll. (b) The bar diagram denotes GUS activities (4- MU Fluorescence) of the different cell types of the leaf midrib from wild type plant (*N. tabaccum* cv Samsun NN, as untransformed control), and transgenic plants generated for the GUS construct: pKYLXGUS (with CaMV35S promoter) and pKMSgt-FSgtGUS (with hybrid promoter). Different cell/tissue type of the leaf midrib is presented. T: Trichomes; UE: Upper epidermis; ChC: Collenchymatous cells; PC: Parenchymatous cells; IP: Internal Phloem; X: Xylem; EP: External Phloem; LE: Lower Epidermis. (c) The bar diagram denotes GUS activities (4- MU Fluorescence) of the different cell types of the stem from wild type plant (*N. tabaccum* cv Samsun NN, as untransformed control), and transgenic plants generated for the GUS construct: pKYLXGUS (with CaMV35S promoter) and pKMSgt-FSgtGUS (with hybrid promoter). Different cell/tissue type of stem is presented. T: Trichomes; E: Epidermal cells; CCl: Cortical collenchymas; CP: Cortical parenchyma; EP: External Phloem; X: Xylem; IP: Internal Phloem; PP: Pith parenchyma. (d) The bar diagram denotes GUS activities (4- MU Fluorescence) of the different cell types of the root from wild type plant (*N. tabaccum* cv Samsun NN, as untransformed control), and transgenic plants generated for the GUS construct: pKYLXGUS (with CaMV35S promoter) and pKMSgt-FSgtGUS (with hybrid promoter). Different cell/tissue type of root is presented. E: Epidermal cells; CC: Cortical cell; X: Xylem; P: Phloem. (e) The bar diagram denotes normalized GUS activities (4-MU Fluorescence) of the MSgt-FSgt promoter in different cell types of stem, leaf midrib, leaf blade and the root of transgenic plants expressing the MSgt-FSgt promoter. T: Trichomes; EP: External Phloem; X: Xylem; IP: Internal Phloem: ChC, Collenchymatous cells; PC: Parenchymatous cells; UE: Upper epidermis; LE: Lower Epidermis; SM: Spongy mesophyll; PM: Palisade mesophyll; SC: Subsidiary cells; GC: Guard cells; CCl: Cortical collenchymas.

The GUS gene expression levels in different cells/tissue types in the leaf, stem and root of tobacco plants transformed with the CaMV35S and MSgt-FSgt promoters were assayed using CLSM as described in the Method section. In the leaf blade, the MSgt-FSgt promoter exhibited 8.57±0.93, 3.29±0.50, 7.57±1.41, 6.48±0.92, 90.2±9.95 and 20.06±2.77 fold higher activity than the 35S promoter in the trichome, lower epidermis, spongy mesophyll, palisade mesophyll, subsidiary and guard cells, respectively (Figure 6a, 6b and 7a and Table 3). The expression acts of the MSgt-FSgt and CaMV35S promoters were also compared in the transgenic leaf midribs using CLSM-based techniques (Figure 6c and 7b). When compared, *GUS* gene expression under control of the hybrid promoter was found to be 7.55 ±1.02, 17.20±2.5, 19.75±2.68, 7.85±1.72, 53.39±4.82, 57.16±8.96, 6.35±0.89, and 41.98±6.45 times stronger in trichomes, collenchymatous cells, parenchymatous cells, upper epidermal cells, lower epidermal cells, internal phloem, xylem and external phloem of leaf midrib, compared to the CaMV35S promoter (Table 3). In the stem, the *GUS* gene expression levels of the MSgt-FSgt promoter were 25.69±2.35, 15.86±3.19, 7.66±1.17 and 15.08±2.48 fold more in trichomes, external phloem, xylem and internal phloem respectively, than the CaMV35S promoter (Table 3, Figure 6d and 7c). In the case of root, the activity of the CaMV35S promoter was undetectable in root epidermal cells, moderate in root cortical cells and in phloem tissue, but exhibited the highest activity in the root xylem tissue. In contrast, the MSgt-FSgt promoter has the capacity to drive the reporter gene expression at a much higher level in all root cell types (Figure 6e and 7d). In summary, the MSgt-FSgt promoter activity was 9.57±1.36, 2.81±0.32 and 6.29±1.31 times stronger in the root cortical cell, xylem and phloem tissues, respectively, compared to the 35S promoter (Table 3). After normalizing the activity of the CaMV35S promoter across the tissues, we observed high level of the MSgt-FSgt promoter expression in xylem (X), external phloem (EP), and internal phloem (IP) of stem; xylem (X) and lower epidermis (LE) of the leaf midrib; guard cell, subsidiary cells (SC) and palisade mesophyll (PM) of the leaf blade and xylem (X), phloem (P) and cortical cell (CC) of root (Figure 7e).

**Table 3:** Comparison of GUS reporter gene expression patterns in different cell types of transgenic plants developed with CaMV 35S and MSgt-FSgt promoters.

Plant parts	Cell types	Ratio of promoter activity obtained from MSgt-FSgt and CaMV 35S promoters (± SD)
**Stem**	Trichomes (T)	25.69±2.35
	External Phloem (EP)	15.86±3.19
	Xylem (X)	7.66±1.17
	Internal Phloem (IP)	15.08±2.48
**Leaf Midrib**	Trichomes (T)	7.55±1.02
	Collenchymatous cells (ChC)	17.20±2.50
	Parenchymatous cells (PC)	19.75±2.68
	Upper epidermal cells (UE)	7.85±1.72
	Lower epidermal cells (LE)	53.39±4.82
	Internal Phloem (IP)	57.16±8.96
	Xylem (X)	6.35±0.89
	External Phloem (EP)	41.98±6.45
**Leaf Blade**	Trichomes (T)	8.57±0.93
	Lower epidermis (LE)	3.29±0.50
	Spongy mesophyll (SM)	7.57±1.41
	Palisade mesophyll (PM)	6.48±0.92
	Subsidiary cells (SC)	90.2±9.95
	Guard cells (GC)	20.06±2.77
**Root**	Cortical cells (CC)	9.57±1.36
	Xylem (X)	2.81±0.32
	Phloem (P)	6.29±1.31

### Comparison of promoter activity of MSgt-FSgt, FS3 and MS8 in vascular tissue

The GUS gene expression patterns in different vascular tissues and cell types of leaves, stems and roots of transgenic tobacco plants carrying the FS3, MS8 and MSgt-FSgt promoters were studied by CLSM. We observed that the MSgt-FSgt promoter was stronger than either of the parent promoters FS3 and MS8 in directing the expression of the transgene (GUS) in all plant vascular tissues and cells. The MSgt-FSgt promoter exhibited 3.09±0.19 and 3.22±0.43 times (Table 4) higher activity than the FS3 and MS8 promoters in xylem tissue of the leaf midrib. Expression of MSgt-FSgt was found to be 4.60±0.12, 2.16±0.78, and 4.72±1.11 times stronger than the FS3 promoter in external phloem, xylem and internal phloem tissues of the plant stem, respectively (Table 4). The MSgt-FSgt promoter was also found to be 4.19±0.42, 2.56±0.32, and 3.88 ±0.32 times stronger than the MS8 promoter in external phloem, xylem and internal phloem tissues (Table 4). Similarly, the MSgt-FSgt promoter was 1.76±0.63 and 2.37±0.87 times stronger than the MS8 promoter in xylem and phloem tissues of the root, respectively. MSgt-FSgt also showed 1.68±0.42 and 2.45±0.61 fold stronger activity than the FS3 promoter in root xylem and phloem tissue (Table 4).

**Table 4:** Comparison of GUS reporter activity in vascular tissues of transgenic plants developed with FS3, MS8 and MSgt-FSgt promoters.

Plant parts	Cell types	Ratio of promoter activity obtained from MSgt-FSgt and MS8 (± SD)	Ratio of promoter activity obtained from MSgt-FSgt and FS3 (± SD)
**Stem**	External Phloem	4.19±0.42	4.60±0.12
	Xylem	2.56±0.32	2.16±0.78
	Internal Phloem	3.88±0.32	4.72±1.11
**Leaf Midrib**	Internal Phloem	0.92±0.17	1.04±0.05
	Xylem	3.22±0.43	3.09±0.19
	External Phloem	0.72±0.03	0.84±0.43
**Root**	Xylem	1.76±0.63	1.68±0.42
	Phloem	2.37±0.87	2.45±0.61

### Nuclear protein binding assay of the MSgt-FSgt hybrid promoter

**Figure 8:**
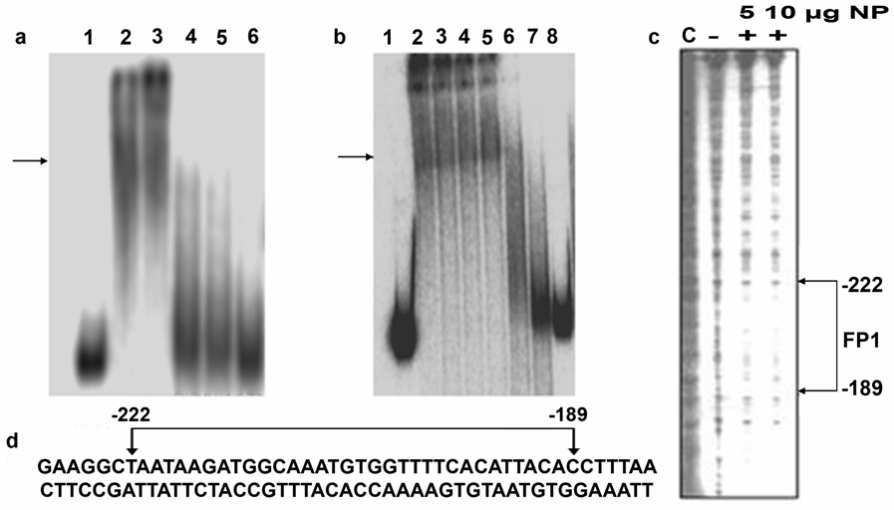
Electrophoretic mobility shift assay (EMSA) and DNaseI foot printing assay to confirm the binding of tobacco nuclear protein to MSgt-FSgt, MS-UAS2 and FS-1promoter DNA. (a) Lane 1, ^32^P-labeled MSgt-FSgt probe only; lane 2–3, ^32^P-labeled MSgt-FSgt probe with 5 µg and 10 µg nuclear protein, EMSA binding is indicated by an arrow; lanes 4–6, describe the competition with 20, 50 and 100 molar excess of unlabeled MSgt-FSgt DNA. (b) Lane 1, ^32^P-labeled MSgt-FSgt probe only; lane 2, ^32^P-labeled MSgt-FSgt probe with 10 µg nuclear protein; lane 3–5, describe competition with 20, 50 and 100 molar excess of unlabeled FS-1 DNA; lanes 6–8, describe competition with 20, 50 and 100 molar excess of unlabeled MS-UAS2 DNA. (c) DNase1 foot-printing assays: The end-labeled MSgt-FSgt promoter was digested in absence (lane marked -) or presence (lane marked +) of tobacco nuclear extract (NP) of 5 and 10 µg protein, and lane marked C refers to sequencing reaction of same DNA fragment in the presence of ddCTP (di-deoxy method for sequencing). The protected DNA sequence is boxed, shown detection of a sequence specific DNA binding from MSgt-FSgt DNA in the presence of tobacco nuclear extracts by DNaseI foot printing analysis. (d) The sequence of the region protected (−222 to −189) from DNaseI digestion is depicted.

The interaction between tobacco nuclear proteins and the MSgt-FSgt promoter DNA was carried out as described in Methods. As shown in Figure 8a, specific DNA-protein binding (marked by an arrow) was observed in the presence of 5 µg and 10 µg of nuclear protein extract. The binding was completely abolished by competition with a 50 fold molar excess of unlabeled MSgt-FSgt DNA indicating that the interaction is specific (Figure 8a). This specific binding was not eliminated by competition with a 100 fold molar excess of FS3 but it was completely abolished by a 100 fold molar excess of the MS8 fragment (Figure 8b).

The promoter sequence where nuclear protein binds was identified by DNaseI foot printing analysis. It was a 33 bp long sequence that stretched between positions −222 to −189 (Figure 8c). A PLACE (http://www.dna.affrc.go.jp/PLACE/) search of the identified sequence, 5′AATAAGATGGCAAA TGTGGTTTTCACATTACAC 3′, (Figure 8d) revealed the presence of an important plant cis-sequence (CANNTG) that represent Myc consensus.

### Analysis of *in vitro* methylation assay of MSgt-FSgt and CaMV35S promoters

**Figure 9:**
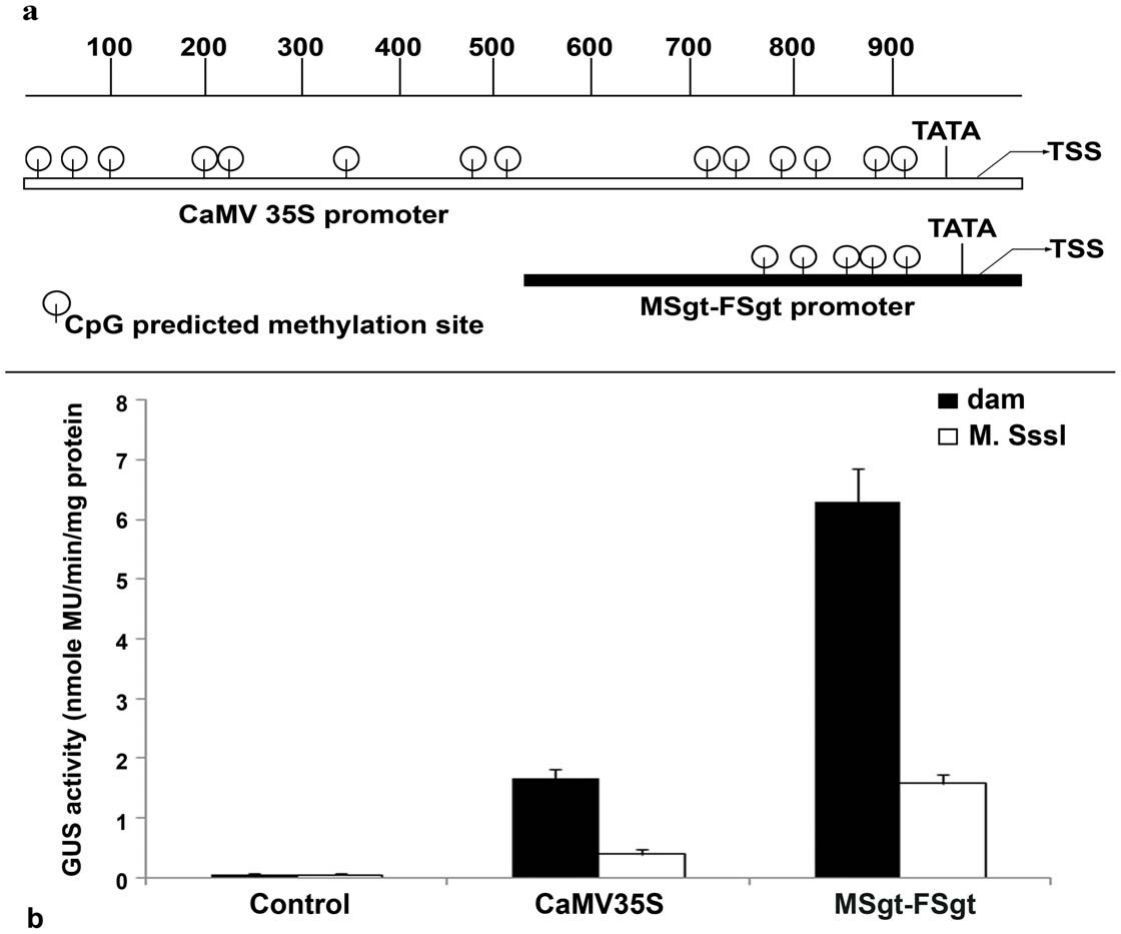
Transient expression analysis of *in vitro* methylated CaMV35S and MSgt FSgt promoter linked with the GUS reporter gene. (a) At the top, the size in bp of the promoter sequence, relative position of TATA box, transcription start site (TSS) in the CaMV35S and MSgt-FSgt promoters are shown. Based on analysis using SVM tool (http://bio.dfci.harvard.edu/Methylator) the putative CG methylation sites were identified and their respective locations were shown. (b) Transient GUS-expression activities of demethylated (dam -ve) and *in vitro* methylated (*M.SssI*) CaMV35S and MSgt-FSgt promoter GUS constructs. The plasmids (pUCPMA-GUS and pUPMSgt-FSgtGUS) were grown in dam^−^ dcm^−^ bacteria and methylated in vitro with *M.SssI* enzyme as described in materials and methods. The data presented is an average ±SD of two experiments, each performed in duplicate, extracts from empty vector (pUCPMA) transformed protoplasts taken as control. The statistical unpaired student t test using Graph Pad Prism (version 5.01) showed significance at *P* value  = 0.0073.

*In silico* analysis of MSgt-FSgt and CaMV35S promoter sequences using software (Support Vector Machine) identified a total number of 5 and 14 CG islands in MSgt-FSgt and CaMV35S promoter respectively (Figure 9a). The transient activities of methylated MSgt-FSgt and CaMV35S promoter in the tobacco protoplast system (Nicotiana tabaccum Xanthi brad) were determined as described in Methods. It was observed that methylated-MSgt-FSgt and CaMV35S promoter showed 64% and 79% less GUS activity compared to unmethylated MSgt-FSgt and CaMV35S promoter respectively (Fig. 9b). Statistical analysis revealed that the decrease in activity in the case of the methylated-MSgt-FSgt promoter (64%) was significant over decrease in the activity of the methylated-CaMV35S promoter (79%). The *P* value obtained was 0.0073 indicating a high level of significance.

## Discussion

Several recombinant promoters were constructed by ligating different up-stream activation sequences of the MS8 promoter to the TATA-containing promoter sequence of the FS3 promoter. One recombinant promoter, MSgt-FSgt, developed in this study showed enhanced activity compared to wild-type promoters and the CaMV35S promoter [44]–[48] in tobacco protoplasts and transgenic tobacco and *Arabidopsis* plants. The observed increase in the relative activities of recombinant promoters may be due to the cooperative combinatorial interactions between the cis-elements present in the up-stream activation sequence (−306 to −125) of MS8 and the TATA element of the FS3 promoter (FS-1). DNaseI foot-printing analysis revealed that a 33 bp long (between the coordinates −222 to −189) nuclear protein binding site (5′AATAAGATGGCAAATGTGG TTTTCACATTACAC3′) may be important for MSgt-FSgt promoter function, because deletion of this portion causes severe loss of MSgt-FSgt promoter activity as evidenced from the differences in activity between the MS-UAS3-FS1 promoter (construct No. 3) and the MS-UAS5-FS1 promoter (construct No. 5) (Figure 1).

In the present study, the MSgt-FSgt promoter showed 10.31 and 14.22 times stronger activity than CaMV35S promoter in the transient protoplast and transgenic tobacco systems respectively. In earlier reports as well as in the present study, it was demonstrated that the parental promoters MS8 [22] and FS3 [23] were 2 to 4 folds stronger than CaMV35S. However, *Mirabilis mosaic virus* full-length transcript promoter (MMV-FLt12) was reported to be 14 times stronger than CaMV35S in tobacco protoplast transient system [29] and about 25 times stronger than the CaMV35S in transgenic tobacco [26]. Based on these observations we may conclude that the MMVFLt12 promoter [26] is stronger than the MSgt-FSgt promoter. However, the recombinant promoter MSgt-FSgt is important because (i) it contains heterogeneous sequences compared to CaMV35S and MMVFLt12 (ii) it directed higher levels of constitutive gene expression in different plant cell types compared to the starting promoters (MS8 and FS3) and CaMV35S (iii) in a separate study by Kumar et al. (unpublished data), it was observed that the MSgt-FSgt promoter showed higher transgene activity under abiotic stress conditions (salt, salicylic/abscisic acid) compared to CaMV35S promoter.

Detailed analysis of GUS-specific transcripts isolated from transgenic plants containing CaMV35S, MS8, FS3 and MSgt-FSgt promoter constructs using Northern blot analysis, real-time PCR and qRT-PCR showed a fair correlation between the steady-state level of mRNA (transcript level) and GUS activity (protein level). Based on the above experimental evidence, it was confirmed that the activity of the MSgt-FSgt promoter was considerably stronger than that obtained from the CaMV35S, MS8 and FS3 promoters. Also it was noted that the MSgt-FSgt promoter directed reporter gene expression more consistently compared to the CaMV35S promoter in two different independent plant systems studied.

The intensities of blue fluorescence obtained from 4-MU (λ_ex_ 363 nm and λ_em_ 460 nm), the hydrolyzed product of MUG in the presence of the GUS enzyme, could be considered to be an indirect measure of relative promoter strength. 4-MU-intensity obtained from a particular cell/tissue type of a transgenic plant harboring the GUS-reporter gene (treated with 1 mM MUG solution) may reflect the cell/tissue specificity of a given promoter. The cell-specific expression of MSgt-FSgt was measured precisely using the analytical software ‘LAS-AF’ supplied with the confocal microscopy system (Leica, Germany), and was compared to that obtained from the MS8, FS3 and CaMV35S promoters. The normalized activity of MSgt-FSgt promoter clearly indicated that it is a near constitutive promoter and stronger than CaMV35S. The value obtained for the control sample (background) may be due to the presence of several candidates for blue fluorescence emissions; those are phenolic substances such as chlorogenic acid, caffeic acid, coumarines, ferulic acid, sinapic acid etc. All of these compounds emit blue fluorescence when excited at 405 nm [49].

The elevated vascular tissue-specific expression of the MSgt-FSgt promoter compared to the two wild-type promoters (FS3 and MS8) and the CaMV35S may be due to the presence of increased numbers of major plant cis-elements like Dof-1 and ARR1 in the MSgt-FSgt promoter in comparison to the wild-type promoters and CaMV35S. Dof-1 plays various roles in plant growth and development [50], [51]. Recent studies showed involvement of Dof-1 in the vascular development of higher plants [52]. ARR1 and ARR2 factors usually act as transcriptional activators that promote the expression of a gene in plant cells through their own target sequences [53]. Besides the Dof-1 and ARR1 elements, the specific distribution pattern of the other cis-elements like Asf-1, G-Box etc. with different intervening spacer sequences in MSgt-FSgt promoter may also contribute to the vascular tissue specific and other functional role of the MSgt-FSgt promoter. Moreover, analyzing the MS8, FS3 and MSgt-FSgt promoter sequence by PlantPAN database [25] other cis-elements like AINTEGUMENTA (ANT), ATHB-9 were also found to be increased in MSgt-FSgt promoter when compared to the two wild-type promoters (MS8 and FS3). Among these, ANT acts as a transcriptional activator playing a critical role not only in regulating ovule and female gametophyte development but also for gene expression in vegetative tissues [54]. The highest activity of MSgt-FSgt hybrid promoter in comparison to FS3 or MS8 in the transgenic system might be due to the increased presence of these cis-regulatory elements.

DNA methylation plays a crucial role in gene silencing [55]. Several studies have provided evidence that the level of gene expression from in vitro methylated DNA is strongly reduced when assayed in animal cells [56]–[59] and in plant cells [60]–[63], and this phenomenon could result in transgene silencing. We observed 64% decrease for MSgt-FSgt promoter is significant over 79% decrease for the CaMV35S promoter (*P* = 0.0073). In stably transformed independent transgenic *Arabidopsis* lines (T_1_ generation; 21 days old), it was also observed that the MSgt-FSgt and CaMV35S promoters resulted 6.7% and 13.3% of transgenic plants with minimum (basal) level of GUS activity respectively. This implies that both promoters are prone to be silenced but the degree of silencing could be higher in the case of the CaMV35S promoter compared to the MSgt-FSgt promoter.

There may be few possible constrains associated with using this promoter in plant genetic engineering. First, this recombinant promoter, being constitutive in nature, lacks tissue specificity. Second, in some situations, we may also need to restrict the expression level of a transgene, otherwise, it may cause problems to the plant itself, being a strong promoter, MSgt-FSgt, may not be ideal for use in such situations.

The observations of the present study revealed a promising future of MSgt-FSgt promoter as a potential candidate promoter for ectopic gene expression in plants. This study also highlighted the usefulness of CLSM in studying quantitative and qualitative gene expression in individual plant cells.

## References

[1] Atchison M (1988). Enhancers: Mechanism of action and cell specificity.. Ann Rev Cell Biol.

[2] Dynan WS (1989). Modularity in promoters and enhancers.. Cell.

[3] Guarente L (1984). Yeast promoters: Positive and negative elements.. Cell.

[4] Guarente L, Yocum R, Gifford P (1982). A GAL10-CYC1 hybrid yeast promoter identifies the GAL4 regulatory region as an upstream site.. Proc Natl Acad Sci USA.

[5] Struhl K (1985). Naturally occurring poly (dA-dT) sequences are upstream promoter elements for constitutive transcription in yeast.. Proc Natl Acad Sci USA.

[6] Bienz M, Pelham HRB (1986). Heat shock regulatory elements functions as an inducible enhancer in the *Xenopus* HSP70 gene and when linked to a heterologous promoter.. Cell.

[7] de Boer HA, Comstock LJ, Vasser M (1983). The tac promoter: a functional hybrid derived from the trp and lac promoters.. Proc Natl Acad Sci U S A.

[8] Bestwick RK, Kellogg JA (2000). Synthetic Hybrid Tomato E4/E8 Plant Promoter - Patent.

[9] Comai L, Moran P, Maslyar D (1990). Novel and useful properties of a chimeric plant promoter combining CaMV35S and MAS elements.. Plant Mol Biol.

[10] Rushton PJ, Reinstadler A, Lipka V, Lippok B, Somssich IE (2002). Synthetic plant promoters containing defined regulatory elements provide novel insights into pathogen- and wound-induced signaling.. Plant Cell.

[11] Last DI, Brettell RIS, Chamberlain DA, Chaudhary AM, Larkin PJ (1991). pEmu: an improved promoter for gene expression in cereal cells.. Theor Appl Genet.

[12] Sawant S, Singh PK, Madanala R, Tuli R (2001). Designing of an artificial expression cassette for the high-level expression of transgenes in plants.. Theor Appl Genet.

[13] Bhullar S, Chakravarthy S, Advani S, Datta S, Pental D (2003). Strategies for development of functionally equivalent promoters with minimum sequence homology for transgene expression in plants: cis-elements in a novel DNA context versus domain swapping.. Plant Physiol.

[14] Ni M, Cui D, Einstein J, Narasimhulu S, Vergara CE (1995). Strength and tissue specificity of chimeric promoters derived from the octopine and mannopine synthase genes.. The Plant J.

[15] Leisner SM, Gelvin SB (1988). Structure of the octopine synthase upstream activator sequence.. Proc Natl Acad Sci USA.

[16] Lee LY, Kononov ME, Bassuner B, Frame BR, Wang K (2007). Novel Plant Transformation Vectors Containing the Superpromoter.. Plant Physiol.

[17] Mol JNM, Stuitje AR, van der Krol A (1989). Genetic manipulation of floral pigmentation genes.. Plant Mol Biol.

[18] Meyer P, Saedler H (1996). Homology dependent gene silencing in plants.. Annu Rev.

[19] Vaucheret H, Fagrad M (2001). Transcriptional gene silencing in plants: targets, inducers and regulators.. Trends Genet.

[20] Chaturvedi CP, Sawant SV, Kiran K, Mehrotra R, Lodhi N (2006). Analysis of polarity in the expression from a multifactorial bidirectional promoter designed for high-level expression of transgenes in plants.. J Biotechnol.

[21] Ye X, Al-Babili S, Kolti A, Zhang J, Lucca P (2000). Engineering the pro-vitamin A (β-carotene) biosynthetic pathways in (carotenoid-free) rice endosperm.. Science.

[22] Dey N, Maiti IB (2003). Promoter deletion and comparative expression analysis of mirabilis mosaic caulimovirus (MMV) sub-genomic transcript (Sgt-) promoter in transgenic plants.. Transgenics.

[23] Bhattacharyya S, Dey N, Maiti IB (2002). Analysis of cis-sequence of subgenomic transcript promoter from the Figwort mosaic virus and comparison of promoter activity with the Cauliflower mosaic virus promoters in monocot and dicot cells.. Virus Res.

[24] Higo K, Ugawa Y, Iwamoto M, Korenaga T (1999). Plant cis-acting regulatory DNA elements (PLACE) database.. Nucleic Acids Res.

[25] Chang WC, Lee TY, Huang HD, Huang HY, Pan RL (2008). PlantPAN: Plant promoter analysis navigator, for identifying combinatorial *cis*-regulatory elements with distance constraint in plant gene groups.. BMC Genomics.

[26] Dey N, Maiti IB (1999a). Structure and promoter/leader deletion analysis of mirabilis mosaic virus (MMV) full length transcript promoter in transgenic plants.. Plant Mol Biol.

[27] Schardl CL, Byrd AD, Benzion G, Altschuler MA, Hildebrand DF (1987). Design and construction of a versatile system for the expression of foreign genes in plants.. Gene.

[28] Jefferson RA, Kavanagh TA, Bevan MW (1987). GUS fusions: beta-glucuronidase as a sensitive and versatile gene fusion marker in higher plants.. EMBO J.

[29] Sahoo DK, Ranjan R, Kumar D, Kumar A, Sahoo BS (2009). An alternative method of promoter assessment by confocal laser scanning microscopy.. J Virol Methods.

[30] Bongaerts RJ, Hautefort I, Sidebotham JM, Hinton JC (2002). Green fluorescent protein as a marker for conditional gene expression in bacterial cells.. Method Enzymol.

[31] Zimmer M (2002). Green Fluorescent Protein: Applications, Structure, and Related photophysical behavior.. Chemical Rev.

[32] Hofgen R, Willmitzer L (1988). Storage of competent cells for Agrobacterium transformation.. Nucleic Acids Research,.

[33] Maiti IB, Murphy JF, Shaw JW, Hunt AG (1993). Plants that express a potyvirus proteinase genes are resistant to virus infection.. Proc Natl Acad Sci USA.

[34] Bradford MM (1976). A rapid and sensitive method for quantitation of microgram quantities of protein utilizing the principle of protein-dye binding.. Anal Biochem.

[35] Zhang X, Henriques R, Lin SS, Niu QW, Chua NH (2006). Agrobacterium-mediated transformation of Arabidopsis thaliana using the floral dip method.. Nature Protocol.

[36] Allen GC, Flores-Vergara MA, Krasnyanski S, Kumar S, Thompson WF (2006). A modified protocol for rapid DNA isolation from plant tissues using cetyl trimethyl ammoniumbromide.. Nature Protocol.

[37] Wu L, Fan J, Jiang L, Wang H, Song R (2003). A specific cis-hairpin ribozyme facilitates infection of a TMV-based DNA vector in tobacco protoplasts.. J Virol Methods.

[38] Livak KJ, Schmittgen TD (2001). Analysis of relative gene expression data using real-time quantitative PCR and the 2^-ΔΔCt^ method.. Methods.

[39] Pfaffl MW (2001). A new mathematical model for relative quantification in real-time RT-PCR.. Nucleic Acids Res.

[40] Escobar C, Aristizabal F, Navas A, Del Campo FF, Fenoll C (2001). Isolation of active DNA-binding nuclear proteins from tomato galls induced by root-knot nematodes.. Plant Mol Biol Reporter.

[41] Lam E, Benfey PN, Gilmartin PM, Fang R-X, Chua NH (1989). Site specific mutations alter in vitro factor binding and change promoter expression pattern in transgenic plants.. Proc Natl Acad Sci USA.

[42] Bhasin M, Zhang H, Reinherz EllisL ,  Reche PedroA (2005). Prediction of methylated CpGs in DNA sequences using a support vector machine.. FEBS Letters.

[43] Pradhan S,  Urwin NigelA R,  Jnkins GarethI,  Adams RogerL P (1999). Effects of CWG methylation on expression of plant genes.. Biochemical Journal.

[44] Benfey PN, Chua N (1990). The cauliflower mosaic virus 35S promoter: combinatorial regulation of transcription in plants.. Science.

[45] Benfey PN, Ren L, Chua NH (1989). The CaMV35S enhancer contains at least two domains which can confer different developmental and tissue-specific expression patterns.. EMBO J.

[46] Benfey PN, Ren L, Chua NH (1990a). Combinatorial and synergistic properties of CaMV35S enhancer subdomains.. EMBO J.

[47] Fang RX, Nagy F, Sivasubramaniam S, Chua NH (1989). Multiple cis regulatory elements for maximal expression of the cauliflower mosaic virus 35S promoter in transgenic plant.. Plant Cell.

[48] Odell JT, Nagy F, Chua NH (1985). Identification of DNA sequences required for activity of the cauliflower mosaic virus 35S promoter.. Nature.

[49] Lang M, Stober F, Lichtenthaler HK (1991). Fluorescence emission spectrum of plant leaves and plant constituents.. Radiat Environ Biophys.

[50] Yanagisawa S (2002). The Dof family of plant transcription factor.. Trends Plant Sci.

[51] Yanagisawa S (2004). Dof domain proteins: plant-specific transcription factors associated with diverse phenomena unique to plants.. Plant Cell Physiol.

[52] Konoshi M, Yanagisawa S (2007). Sequential activation of two Dof transcription factor gene promoters during vascular development in Arabidopsis thaliana.. Plant Physiol Biochem.

[53] Sakai H, Aoyama T, Oka A (2000). Arabidopsis ARR1 and ARR2 response regulators operate as transcriptional activators.. Plant J.

[54] Klucher KM, Chow H, Reiser L, Fischer RL (1996). The AINTEGUMENTA gene of arabidopsis required for ovule and female gametophyte development is related to the floral homeotic gene APETALA2.. THE PLANT CELL.

[55] Ingelbrecht Ivan, Van Houdt H, Van Montagu M, Depicker A (1994). Posttranscriptional silencing of reporter transgenes in tobacco correlates with DNA methylation.. Proc Natl Acad Sci USA.

[56] Keshet I, Yisraely J, Cedar H (1985). Effect of regional DNA methylation on gene expression.. Proc Natl Acad Sci USA.

[57] Gotz F, Schulze-Forster H, Wagner H, Kroger H, Simon D (1990). Transcriptional inhibition of SV40 by in vitro DNA methylation.. Biochim Biophys Acta.

[58] Levine A, Cantoni GL, Razin A (1991). Inhibition of promoter activity by methylation possible involvement of protein mediators.. Proc Natl Acad Sci USA.

[59] Hsieh C (1994). Dependence of transcriptional repression on CpG methylation density.. Mol Cell Biol.

[60] Hershkovitz M, Gruenbaum Y, Renbaum P, Razin A, Loyter A (1990). Effect of CpG methylation on gene expression in transfected plant protoplasts.. Gene.

[61] Weber H, Ziechmann C, Graessmann A (1990). In vitro DNA methylation inhibits gene expression in transgenic tobacco.. EMBO J.

[62] Hohn T, Corsten S, Rieke S, MuÈ ller M, Rothnie H (1996). Methylation of coding region alone inhibits gene expression in plant protoplasts.. Proc Natl Acad Sci USA.

[63] Dieguez MJ, Bellotto M, Afsar K, Mittelsten Scheid O, Paszkowski J (1997). Methylation of cytosines in nonconventional methylation acceptor sites can contribute to reduced gene expression.. Mol Gen Genet.

